# Chylothorax After Schwannoma Resection

**DOI:** 10.7759/cureus.30112

**Published:** 2022-10-09

**Authors:** Henry Zou, Behrooz Shabahang

**Affiliations:** 1 Thoracic Surgery, Michigan State University College of Human Medicine, Grand Rapids, USA; 2 Thoracic Surgery, Trinity Health, Grand Rapids, USA

**Keywords:** chemical pleurodesis, thoracic duct ligation, chylothorax, resection, schwannoma

## Abstract

Schwannomas are tumors derived from Schwann cells of the peripheral nerve sheath that are usually benign; nonetheless, they can cause significant morbidity. When indicated, surgical resection is the gold standard of treatment for schwannomas. However, chylothorax is a rare postoperative complication of thoracic surgery. We present a case of chylothorax after thoracic schwannoma resection. A 61-year-old woman underwent a computed tomography (CT) scan for suspected nephrolithiasis, which instead found a right mediastinal mass that was confirmed to have features consistent with a schwannoma on thoracic spine magnetic resonance imaging (MRI). Right thoracotomy and schwannoma resection were performed, resulting in the complete removal of the schwannoma without capsular invasion. Two chest tubes were also inserted. On postoperative day 1 (POD1), the patient presented with a chylothorax that was initially treated with chest tube suctioning and total parenteral nutrition (TPN). However, a repeat right thoracotomy with thoracic duct ligation and dry talc chemical pleurodesis was subsequently performed on POD15 due to a lack of clinical improvement, which saw the resolution of the chylothorax without recurrence. Chylothorax is a rare but severe postoperative complication of thoracic surgeries, including those that involve tumor resections. We present a case of chylothorax after thoracic schwannoma resection that initially failed conservative management but eventually resolved after thoracic duct ligation and chemical pleurodesis. This case highlights the need for effective non-surgical treatments for chylothorax, the importance of remaining vigilant for rare postoperative complications, and the need for randomized controlled trials (RCTs) to develop a standardized chylothorax management algorithm.

## Introduction

Schwannomas, also called neurilemmomas, are encapsulated tumors derived from neoplastic Schwann cells of the peripheral nerve sheath [1]. They occur most often as a single, sporadic lesion and are the most common peripheral nerve tumors (PNTs) in adults [1]. Though they have low proliferative potential and extremely low rates of malignant transformation, schwannomas can cause significant morbidity secondary to mass effects on critical anatomical structures or bone erosion [2]. Schwannomas are usually asymptomatic but can present with dysesthesia, sensory loss, weakness, and radicular pain [1,2]. They can be diagnosed through magnetic resonance imaging (MRI), computed tomography (CT), ultrasound, and histology [1]. As schwannomas often grow too slowly to respond to chemotherapy, surgical resection is considered the gold standard treatment for symptomatic and high-risk cases [2]. Chylothorax, or the accumulation of chyle-containing lymphatic fluid from the thoracic duct in the pleural space, is a rare postoperative complication of thoracic surgery that is associated with high morbidity and mortality if left untreated [3]. Chylothorax can be treated conservatively with total parenteral nutrition (TPN), somatostatins, and therapeutic thoracentesis or surgically via thoracic duct ligation and/or pleurodesis [3]. We present a case of postoperative chylothorax occurring after right-sided flexible bronchoscopy, bronchioloalveolar lavage, thoracotomy, and schwannoma resection.

## Case presentation

A 61-year-old woman with a history of obstructive sleep apnea, hypertension, obesity, gastric bypass surgery, anemia, atypical ductal breast hyperplasia, and arthritis reported gross hematuria and underwent CT scans for evaluation of nephrolithiasis. Though no stones were identified, a mediastinal mass was noted in the right lower chest. Subsequent thoracic spine MRI showed a well-circumscribed 10 cm paravertebral mass consistent with schwannoma. Following consultation and cardiac clearance, a right thoracotomy and schwannoma resection were scheduled in three weeks.

On the scheduled date, flexible bronchoscopy, bronchioloalveolar lavage, right posterolateral thoracotomy, and schwannoma resection at the T8-T9 level were performed. Bronchoscopy was used to evaluate for potential airway compression, and bronchioloalveolar lavage was used to assess for potential malignant cells in the lower respiratory tract. The entire schwannoma was removed as an R0 resection. There was no evidence of capsular invasion, and the intercostal nerves were left undisturbed. Resection of a lymph node posterior to the schwannoma was also performed, and a 28-French (28F) angled chest drain was inserted in the posterior gutter while a 28F straight chest drain was inserted toward the apex. No perioperative complications were reported, and the patient was taken to the post-anesthesia care unit (PACU) in stable condition. Postoperative chest X-rays (CXRs) showed mild bilateral atelectasis but no pneumothorax.

On postoperative day 1 (POD1), the patient presented with chylothorax. Chest tubes were used to suction drainage, the patient was placed on a clear liquid (CL) diet, and she was monitored via recurrent complete blood counts (CBCs), complete metabolic panels (CMPs), and CXRs. On POD4, the CL diet was discontinued as it contributed to excess chest tube drainage. A "nothing by mouth" (NPO) protocol with the exception of medications was initiated, and a peripherally inserted central catheter (PICC) line was placed for TPN treatment. From POD5-POD8, the serous drainage remained high. Meanwhile, CMPs indicated hypomagnesemia and hyperphosphatemia. Thus, on POD6, magnesium and calcium were added to the TPN regimen while the phosphorus dose was decreased. On POD9, the serous drainage continued to remain high. Little changes in the amount of drainage were observed from POD9-POD14, so a repeat flexible bronchoscopy and right thoracotomy with thoracic duct ligation were scheduled.

**Figure 1:**
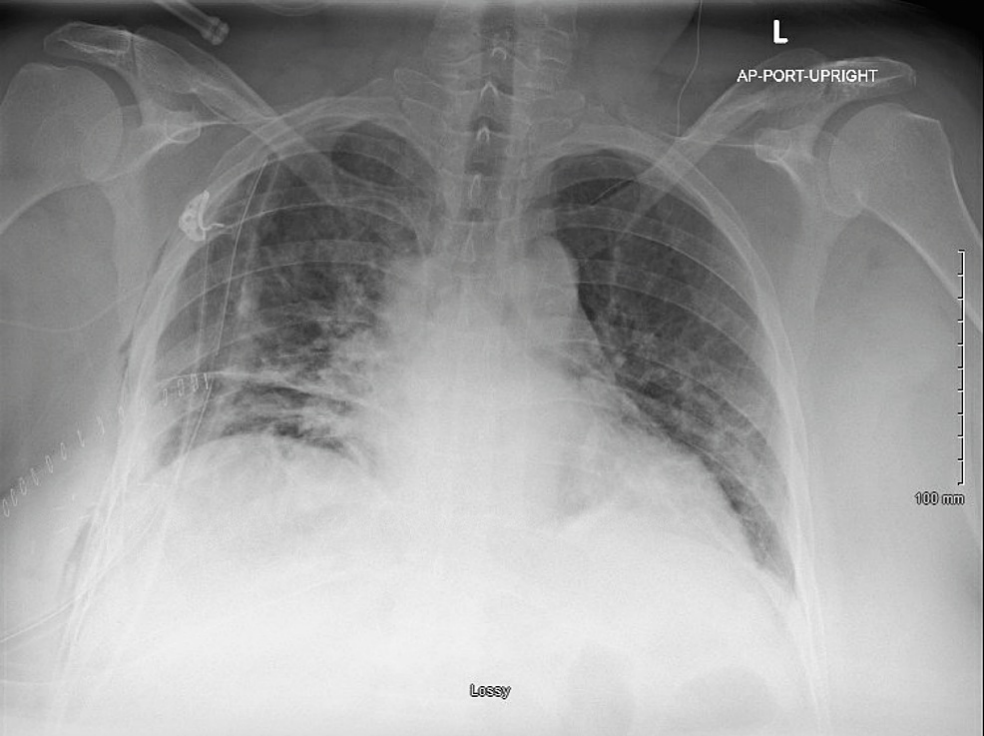
Resolution of chylothorax after thoracic duct ligation and chemical pleurodesis L: left, AP: anteroposterior, PORT: portable, Lossy: some data lost during image compression

On POD14, a flexible bronchoscopy, bronchioloalveolar lavage, repeat right thoracotomy, adhesion takedown between the visceral and parietal pleura, thoracic duct ligation, application of a dural seal on the suture ligation, and dry talc chemical pleurodesis were performed (Figure 1). Furthermore, the chest tubes were replaced. No perioperative complications were reported, and the patient was sent to the PACU in stable condition.

The patient underwent resolution of chylothorax after thoracic duct ligation was attempted on POD15 by right thoracotomy approach; subsequently, TPN was discontinued and a regular diet was resumed. Additionally, the chest tubes were changed to water-sealed. For the next two days, drainage was low with no milky output. On POD18, the chest tubes were removed. On POD20, bilateral lower extremity (BLE) edema was noted, so furosemide and hydrochlorothiazide were initiated. On POD21, the patient’s staples were removed while supplemental potassium chloride was increased for the BLE edema; the patient’s potassium levels were also monitored daily. Electrolyte management continued for two days, and the patient was discharged on POD24 for outpatient follow-up.

## Discussion

Chylothorax is a rare postoperative complication of thoracic surgery, including thoracic tumor resection [4]. In a 21-year analysis of 203 cases of chylothorax at the Mayo Clinic, 48 cases (25%) were attributed to surgical etiologies; among these, nine cases (4.4%) were attributed to mediastinal mass resections [4]. A recent study of 31 patients treated surgically for giant mediastinal tumors reported one postoperative case of chylothorax, which was successfully treated using fasting and a three-day course of somatostatins [5].

Surgical resection remains the mainstay and gold-standard therapeutic option for schwannomas; in a study of 291 patients presenting with sporadic peripheral schwannomas, 232 of them (80%) underwent resection [2]. Among these surgical patients, only two patients (0.009%) experienced postoperative chylothorax [2]. Similarly, in a study of 80 patients who underwent surgical resections of intrathoracic neurogenic tumors (of which 52 were schwannomas/neurilemmomas), only one patient presented with postoperative chylothorax that was treated conservatively [6]. 

Chylothorax is commonly caused by damage or rupture of the thoracic duct (Table 1), which can induce a rapid accumulation of lymphatic fluid in the pleural space [3,4]. Iatrogenic trauma can induce chylothorax, with thoracic surgery as the leading cause. However, malignant and benign tumors can also cause chylothorax by damaging or obstructing the thoracic duct [3,4]. Another less common cause of chylothorax is mediastinal lymphadenopathy, which can induce extravasation of lymphatic fluid into the pleural space by compressing lymphatic vessels and preventing lymph drainage from the lung periphery [3].

**Table 1:** Common etiologies of chylothorax (not exhaustive)

Traumatic		Atraumatic	
Iatrogenic	Non-iatrogenic	Malignant	Non-malignant
Thoracic and cardiovascular surgery	Penetrating or nonpenetrating trauma to the neck, thorax, or upper abdomen	Lymphoma	Benign tumors
Head and neck surgery	Childbirth	Mediastinal or primary lung malignancies	Lymphangioleiomyomatosis
Spine surgery	Forceful coughing or emesis	Metastatic extrathoracic malignancies	Sarcoidosis
Radiation	Stretching	Sarcoma	Amyloidosis
Transabdominal vagotomy	Yawning	Leukemia	Hemangiomatosis

In our case, chylothorax could be attributed to thoracic duct damage from the thoracic schwannoma resection or the schwannoma itself. Moreover, the enlarged mediastinal lymph node that was also resected may have contributed to the chylothorax by independently inhibiting lymphatic drainage or damaging the thoracic duct. Nonetheless, chylothorax has a significant potential for morbidity and mortality as it can mediate malnutrition via loss of proteins, fats, and vitamins; immunosuppression via loss of immunoglobulins and T-cells; and dyspnea and/or respiratory distress due to pleural effusion [3].

Management of chylothorax is largely dependent on the volume of drainage [3]. Low-output chylothorax (drainage <1 L/day) is usually treated conservatively with somatostatins/octreotide/TPN/chest tubes, while high-output chylothorax (drainage >1 L/day) is usually treated aggressively with surgical interventions including thoracic duct ligation, clipping of thoracic duct tributaries, and chemical pleurodesis [3,7]. However, given the rarity of chylothorax incidence, management of this urgent complication is challenged by a lack of high-quality randomized controlled trials (RCTs) that can generate a universal management algorithm [7]. In a case of chylothorax after surgical resection of a paravertebral ganglioneuroma in a 17-year-old boy, chest tubes, intravenous octreotide, TPN, direct thoracic duct ligation, and pleurodesis all failed to reduce drainage, which reached 0.9-1.2 L/day [8]. Ultimately, only sealing the suspected sources of leakage over the site of the surgical sutures in the left paravertebral sulcus using fibrin sealant patches led to the resolution of the chylothorax [8]. Another case of chylothorax after thoracic schwannoma resection in a 40-year-old man saw drainage of 1.5 L/day but saw successful resolution on fasting, TPN, and subcutaneously-administered somatostatin after 40 postoperative days [9]. Finally, a chylothorax after right thoracic schwannoma resection in a 66-year-old woman saw drainage >1 L/day and failed chest tube drainage and fasting [10]. However, the chylothorax resolved after ligation of the thoracic duct and site of the chyle leak via clipping followed by pleurodesis [10]. In our case of high-output chylothorax, we initially attempted chest tube drainage and TPN, but ultimately achieved a resolution without recurrence through thoracic duct ligation and dry talc chemical pleurodesis after conservative management failed.

## Conclusions

Although surgical resection is the gold standard treatment for schwannomas, chylothorax is a rare but severe complication of thoracic surgery, including those involving tumor resection. We present a case of chylothorax after thoracic schwannoma resection that failed conservative management and ultimately resolved following thoracic duct ligation and dry talc chemical pleurodesis. This case demonstrates the need for effective non-surgical treatment options for chylothorax, particularly for patients who are not surgical candidates. Moreover, this case highlights the importance of remaining vigilant for rare postoperative complications such as chylothorax and the urgent need for well-conducted RCTs that would enable the development of a reliable universal management algorithm for chylothorax.
